# Supplementary material for: Chromothripsis during telomere crisis is independent of NHEJ, and consistent with a replicative origin
Source: Genome Res. 2019 May;29(5):737–49. doi: 10.1101/gr.240705.118 (PMC6499312; doi:10.1101/gr.240705.118)
Supplement: Supplemental Material [file supp_gr.240705.118_Supplemental_file_1.zip › contigs/annotated_contigs/DB57/contig.6.DB57_length_1378_mean_cov_18.9622641509.docx]

**DB57_length_1378_mean_cov_18.9622641509**

```
TTAAATAGAATTCTAGTATCTCGGGTAGGAAATTTATTACAGGCAACCCTAAGAAGATATATTCCCTAAACTCTCCTTCAGGTGATGGG
 >chr7:146142970-146143509 + E=6e-306
ATTAATCTTGGTAAAGTGACTAAGTTGCTTCTTGATGGTTGGTACATTTTGAAAAAGAAGAATCACATTAACTCCAGAGCAGAAAAGAA

CCGTAGATCATTCTTACTAAGTTCATTGGTCTTACATGTAAATTGAGAAAGATTGCTGAAGGCAAGATTGCCCCAAGGAATTTAACTTG

AAATCAAGAGGCTACATAAATGTAAAGTGCTACTGCTTAAATAAAAAATATTATGTTTTAATAATAATCATGATAATATGACACAGTAT

GAAGCAAATATAGTGAAAACTAGAAAACAATCTTGGAAACAGTTGCTACATTGTTATTAAGGACTAAACCAATTTAGAGGATCGTGGCT

GTGAACATTTTAATATCCCACTCAGCTCCAAATTATCTAGAGAAATCTTCTACACATTCTACAATATGGAATGCCCAAGGCTGAGGGAG

TTG|CA|AAACAAAACAAAACAAAAAATCTGTCCTATATTAACATAAGTATATTAAAATTTGACAGACCAGTTGCTGTACTTTTTGGAT
    >chr7:146137219-146137344 + E=4e-63 p=1e-02
TGGTTTTTGAAAACATAGACTTTAGTAGTAAGAAACTTAGT|CTAAAAA|AAACTTAGTGGAGTAAAAGTAAATGAAGATGAGTACTTT
                                                 >chr7:146137335-146137406 + E=1e-30 p=
TAAAATTTAAAGTGTGGTCAGTTAAAACTT|AT|CTTCATTTACTTTTACTCCACTAAGTTTCTTACTACTAAAGTCTATGTTT|ATTA
2e-02                          >chr7:146137313-146137365 - E=3e-19                  >c
AAAGTGAATAAGAGGTACAAAGATGGAAAATCAGTAATGGTTATTGCTCACCAGTCAGGTCTGTCCCATTCCACCATAGCCATGATCTC
hr7:147363870-147364078 + E=6e-113
AAAGAATGAGAACAAAATGATGGAAGCTGAAAAGATCTTCTTCATTGAAGGCAATGAGACTAACAACAGTTCAGGAAGAGCCTATATCA

GATATGGAGAAACTTCTAATGACCTG|TAAATGACCAG|GTGTCAGATAACTGAAGTAAATGGTATTTAACAGCATGGGGTTCCAATAA
                                      >chr7:146052424-146052793 + E=9e-210
CACAGGAGAACACAGGAGCAACCTTCTAGGCTTTATTTCATTCAAGTCCATAAAGTCCCCTGTGGTCGCAGCAGTGCATATTTAAAATC

AGTTAATCATGTTACTCATGGGTGTTGGATATCTTTCAAAATGGCCTCTGGTAATGAGCTATTTGTCCTGCATTGTGACAATAAGTCTC

TTCCATAATTTATAAAGCTCATGCAGATGATGGTTAGAGATTCCATAAGAAAATAATGCAGGGATGTTCTACTTCCTGCTTAATTCCTA

CTTAAATACAGTTAGATACTATAAGTGCATAAATCTACATAGTCCACAATAC
```
